# Case Report: Rectal perforation combined with presacral infection following rubber band ligation and sclerotherapy for hemorrhoids

**DOI:** 10.3389/fmed.2026.1938366

**Published:** 2026-10-14

**Authors:** Yanqing Lu, Ziwen Wang, Zhenfu Shi, Zhi Jiang, Li Zhang, Guolian Gan, Zhen Zhou, Hao Wang

**Affiliations:** 1 The Second Clinical Medical College of Guangzhou University of Chinese Medicine, Guangzhou, China; 2 Department of Coloproctology, The Affiliated Guangzhou Hospital of TCM of Guangzhou University of Chinese Medicine, Guangzhou, China; 3 Department of Proctology, Yinchuan Hospital of Traditional Chinese Medicine Affiliated to Ningxia Medicine University, Yinchuan, China; 4 Department of Colorectal Surgery, The Second Affiliated Hospital of Guangzhou University of Chinese Medicine, Guangzhou, China

**Keywords:** anal stenosis, case report, fecal diversion, injection sclerotherapy, presacral infection, rectal perforation, rubber band ligation

## Abstract

**Background:**

Currently, rubber band ligation and injection sclerotherapy represent the first-line therapeutic strategy for grade I–III internal hemorrhoids. As an invasive procedure, it may still cause postoperative complications, among which, rectal perforation is one of the most serious complications.

**Case presentation:**

This paper describes a patient treated at our facility who experienced a rectal perforation and presacral infection following rubber band ligation and injection sclerotherapy. In the first stage, we performed laparoscopic double-barreled transverse colostomy combined with anal irrigation and anal dilatation to achieve fecal diversion and adequate drainage of the infectious focus. The patient achieved a satisfactory postoperative recovery. Stoma reversal was performed 6 months after surgery.

**Conclusion:**

Rectal perforation combined with presacral infection is a rare but severe complication of rubber band ligation and sclerotherapy for hemorrhoids, among these, sclerotherapy is the primary factor, while rubber band ligation is a contributing factor. It is mostly related to inappropriate iatrogenic operative manipulation. Clinically, strict standardization of rubber band ligation and injection sclerotherapy procedures is required, and close surveillance of high-risk patients after surgery should be strengthened to avoid complications and prevent delayed diagnosis and intervention.

## 1 Introduction

Rectal perforation is mostly iatrogenic trauma. The intestines occupy a large portion of the abdominopelvic cavity and are adjacent to multiple visceral organs. Urological and gynecological surgery are the most common etiological factors. Improper separation and thermal injury during operation are both prone to inducing rectal perforation (1). Sclerotherapy is generally regarded as a relatively safe invasive procedure, with pain and bleeding as its common complications (2). Misplacement of the injection may result in mucosal ulceration or necrosis, and rare complications such as abscess and sepsis (3). Rectal perforation following rubber band ligation (RBL) is a relatively rare clinical complication. A review including 39 studies with a total of 8,060 patients receiving RBL demonstrated an overall complication rate of 14%, with infection accounting for 0.05% and anal fissure combined with anal fistula for 0.4% (4). Presacral infection represents a secondary complication of rectal perforation (5). The rectum lies in intimate anatomical continuity with the presacral space; when the rectal wall is damaged, intestinal contents flows along the retroperitoneal plane toward the presacral compartment. Due to the absence of a natural outlet and the loose tissue structure, these contents accumulate in this area, making it highly susceptible to infection, may further evolve into necrotizing fasciitis or cellulitis (6). Herein, we describe the clinical course, diagnosis, surgical technique and follow-up outcomes of this rare case to provide guidance for similar cases.

## 2 Case report

A 46-year-old female patient, without underlying comorbidities and history of anal surgery, arrived with a 2-year history of circumferential prolapse of anal masses upon feces that required manual reduction and gradually got worse. One day prior to admission, there was severe swelling and agony along with prolapsed anal masses that could not be reduced after feces. Physical examination revealed circumferential hemorrhoidal prolapse at the anal margin with edema and thrombosis; anoscopy showed no congestion or edema of the rectal mucosa and no mucosal congestion or folding upon withdrawal. She was diagnosed with mixed hemorrhoids with incarceration. On October 2, 2025, she underwent external hemorrhoidectomy combined with rubber band ligation and sclerotherapy under spinal anesthesia at a local hospital. Postoperatively, she developed a fever peaking at 39.5 °C, along with difficult defecation, intense anal pain during defecation, perianal swelling, and scant purulent discharge from a small perianal ulcer. She was admitted to the same local hospital for further management, where a diagnosis of “anal sphincter achalasia” was made, and she received symptomatic treatment, including antibiotic and analgesic therapy; however, symptoms failed to achieve significant relief.

On November 5, 2025, she was accepted to the Department of Colorectal Surgery, the Second Affiliated Hospital of Guangzhou University of Chinese Medicine. Physical examination revealed scattered, incompletely healed surgical scars around the anus. An external opening was found 2 cm anterolaterally on the left perianal area with mild erythema of the surrounding soft tissues. Slight purulent discharge exuded upon compression, and a cord-like tract was palpable beneath the skin. Digital rectal examination reveals moderate anal stenosis. Pelvic CT revealed edema and thickening of the wall of the distal rectum and anal canal, with a slightly hypodense presacral shadow and free gas communicating with the distal rectum and anal canal. Pelvic magnetic resonance imaging (MRI) (Figure 1) revealed discontinuity of the lower rectal wall at the 7–8 o’clock position, which was strongly suggestive of rectal perforation. Thickening and pneumatosis were detected in the soft tissues around the lower rectum and anal canal as well as in the presacral space, and an arc-shaped infectious lesion was identified within the intersphincteric space of the internal and external anal sphincters. Colonoscopy (Figure 2) displayed postoperative manifestations after hemorrhoid surgery, and a fistulous opening was identified in the lower rectum.

**Figure 1:**
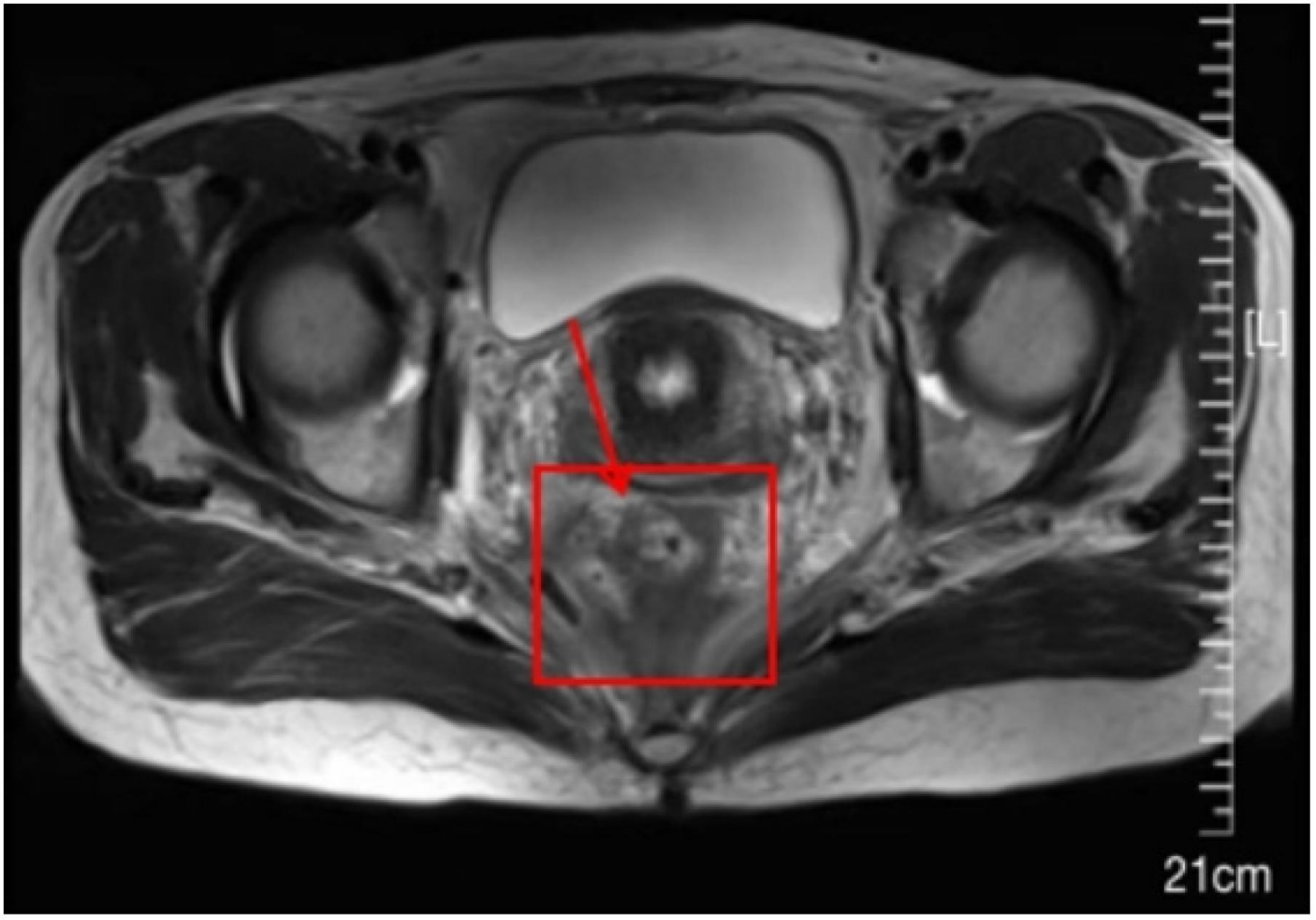
Preoperative pelvic MR image (transverse plane), the arrow indicates a discontinuity in the rectal wall, and the rectangular indicates the presacral infection.

**Figure 2:**
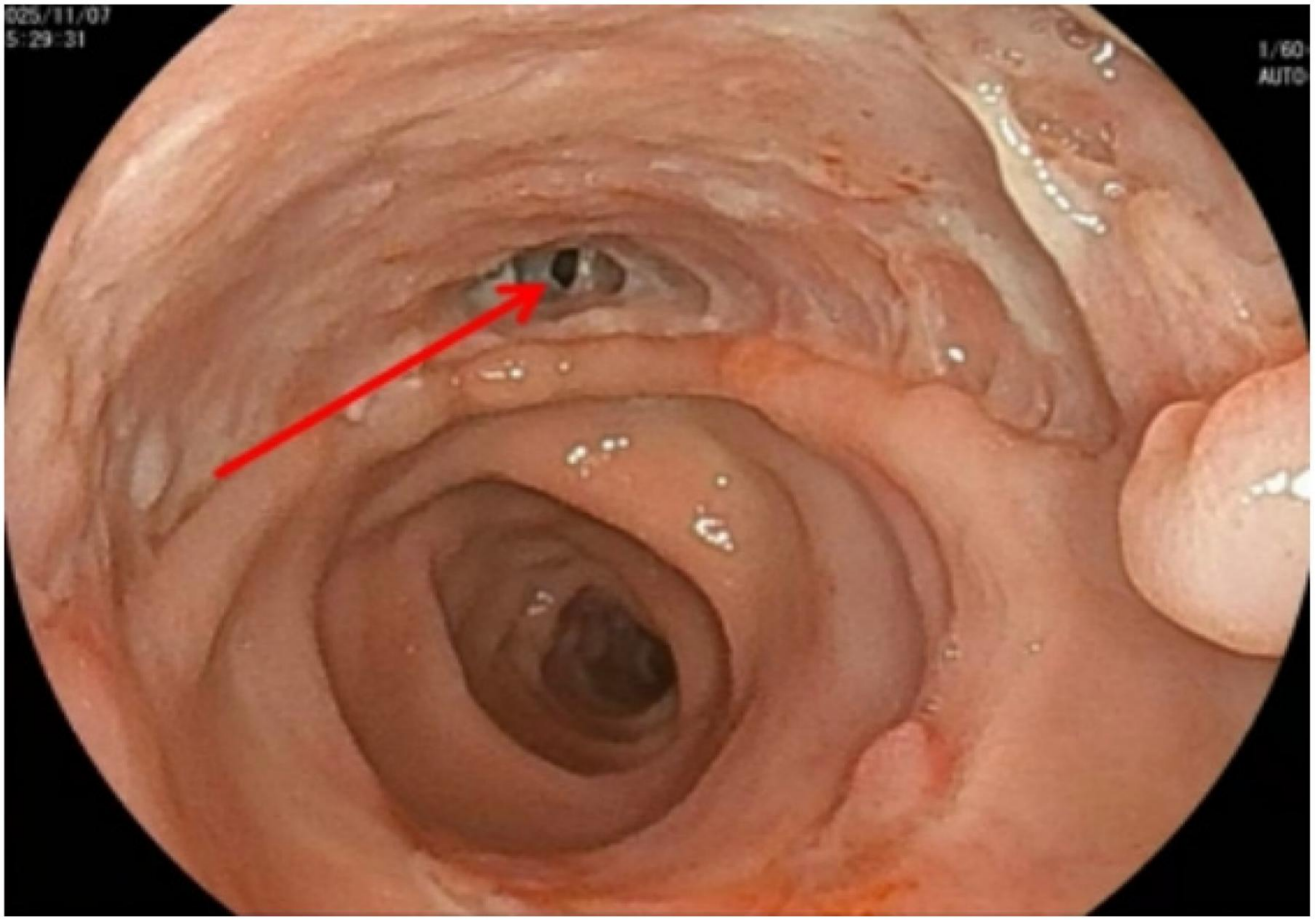
Preoperative colonoscopy image, shows a fistula tract; the arrow indicates the location of the fistula tract.

Combining medical history, physical examination and auxiliary examinations, a final diagnosis of rectal perforation complicated by presacral infection was established. Symptomatic interventions including antibiotic therapy and defecation control were administered, yet the clinical response was unsatisfactory. Given the extensive range of the presacral infection, simple conservative antimicrobial treatment was insufficient to control the inflammatory process. We consulted 12 domestic authoritative experts in colorectal surgery, and it was determined that the patient presented with four key issues requiring targeted management: moderate anal stenosis, presacral infection, rectal fistula, and long-term rectal stricture secondary to rectal mucosal defects. After communicating the patient's condition with her family and conducting a departmental group discussion, the following therapeutic principles were formulated: diverting fecal flow to block the source of infection, performing anal irrigation to control infection, and implementing anal dilatation to relieve anal stenosis.

On November 11, 2025, the patient underwent laparoscopic double-barrel transverse colostomy under endotracheal intubation anesthesia. Intraoperative exploration revealed extensive peritoneal adhesions between the omentum and transverse colon, the omentum and abdominal wall, and the omentum and small intestine. No obvious purulent effusion or signs of diffuse peritonitis were observed in the abdominal cavity. A large volume of normal saline was administered for anal intraoperative irrigation until the effluent turned clear. The transverse colon was exteriorized through the abdominal incision and fixed with circumferential sutures to form a double-barrel stoma; the stoma was confirmed to admit two fingers in diameter. One pelvic drain was placed in the right pelvic cavity. The operation proceeded uneventfully, with an operative duration of 95 min and an estimated intraoperative blood loss of 5 mL. Postoperatively, symptomatic treatments including antibiotic therapy and nutritional support were continued. On postoperative day 3, gas discharge and a small amount of watery stool were observed from the stoma. On postoperative day 5, the patient was in stable condition with satisfactory general status. The pelvic drain was removed; abdominal incision pain was relieved, and gas and stool passed smoothly through the stoma. The abdominal dressing remained dry and clean, and the patient was discharged from the hospital.

During the outpatient follow-up period, the patient self-administered anal dilation using an anal dilator and anal irrigation, in combination with enema therapy using a traditional Chinese medicinal Ilex pubescens enema solution. At 2 months postoperatively, repeat pelvic MRI (Figure 3) demonstrated that the fistulous opening had significantly decreased in size compared with the previous findings, and the infectious lesions had been markedly absorbed. Consequently, the above-mentioned treatment regimen was continued. At 4 months postoperatively, digital rectal examination revealed no significant stenosis, and anoscopy showed that the wounds in the anal canal had healed. To evaluate the therapeutic response to antibiotic treatment and determine whether the patient was eligible for colostomy reversal, repeat a pelvic MRI (Figure 4) was performed. The imaging results showed the reduction of fistula tract and improved resolution of infectious lesions compared with prior scans. At 6 months postoperatively, repeat pelvic MRI (Figure 5) demonstrated complete disappearance of the fistulous opening and most resolution of infectious lesions. Defecography (Figure 6) showed no contrast agent leakage. Colonoscopy confirmed complete local healing (Figure 7), and consequently, colostomy reversal was performed. The postoperative course was uneventful. During follow-up to date, the patient maintained normal bowel movements and reported no significant discomfort.

**Figure 3:**
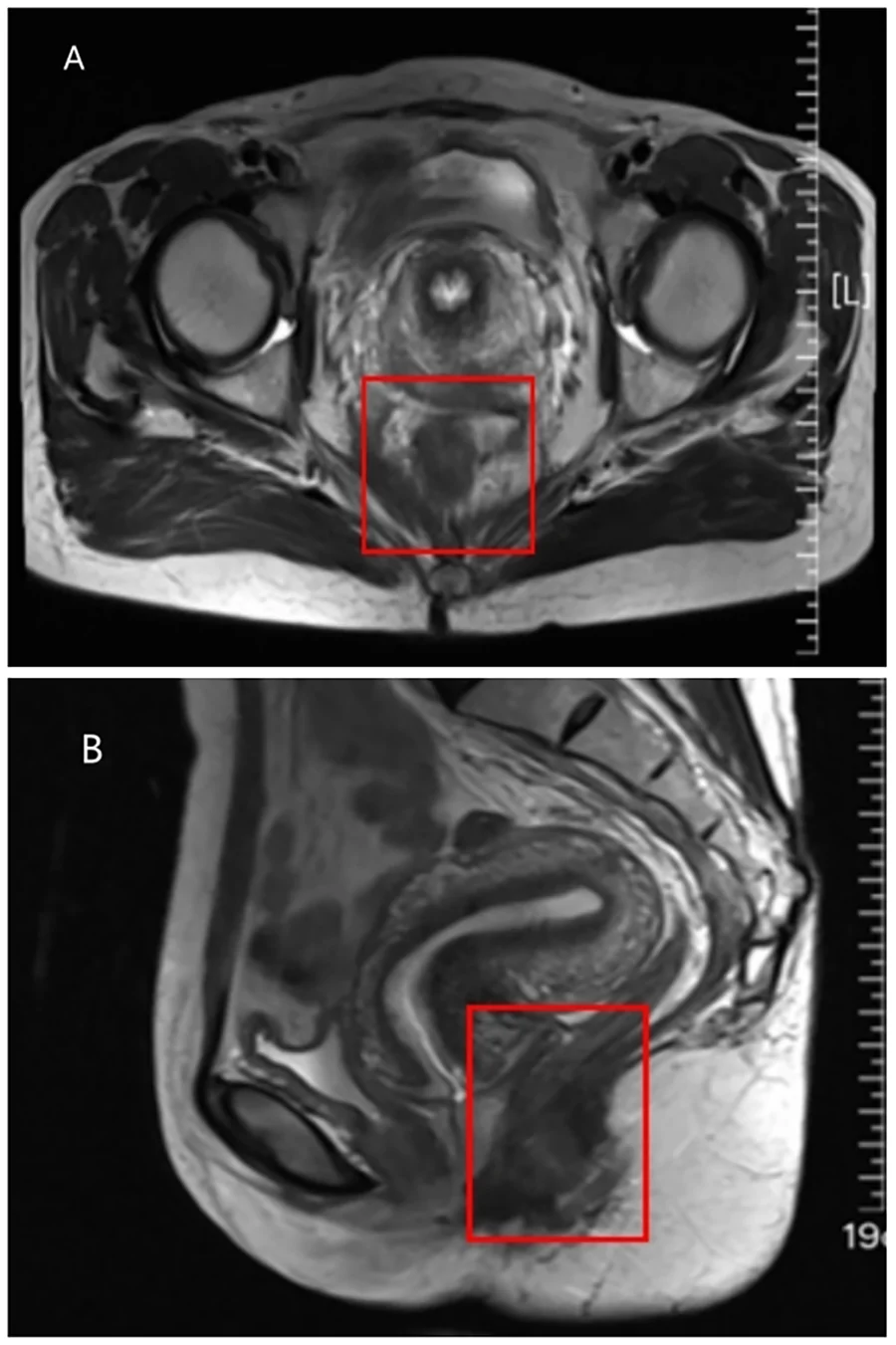
Pelvic MR images at 2 months after surgery (**A** is transverse plane and **B** is sagittal plane), compared to Figure 1, the fistula tract has narrowed, and the infection has resolved.

**Figure 4:**
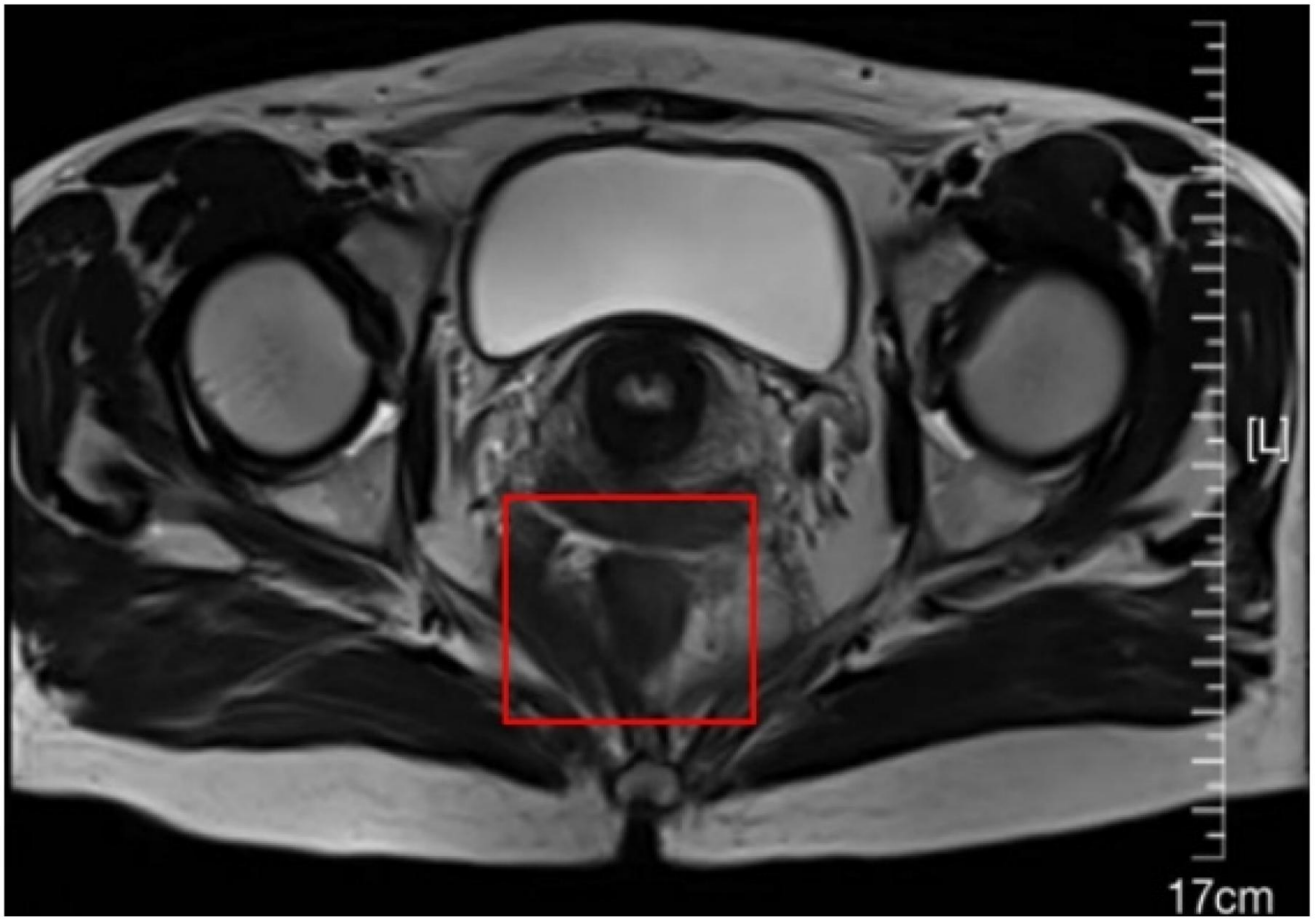
Pelvic MR images at 4 months after surgery (transverse plane), compared to Figure 3, the fistula tract has narrowed, and the infection has resolved. But it has not yet fully healed.

**Figure 5:**
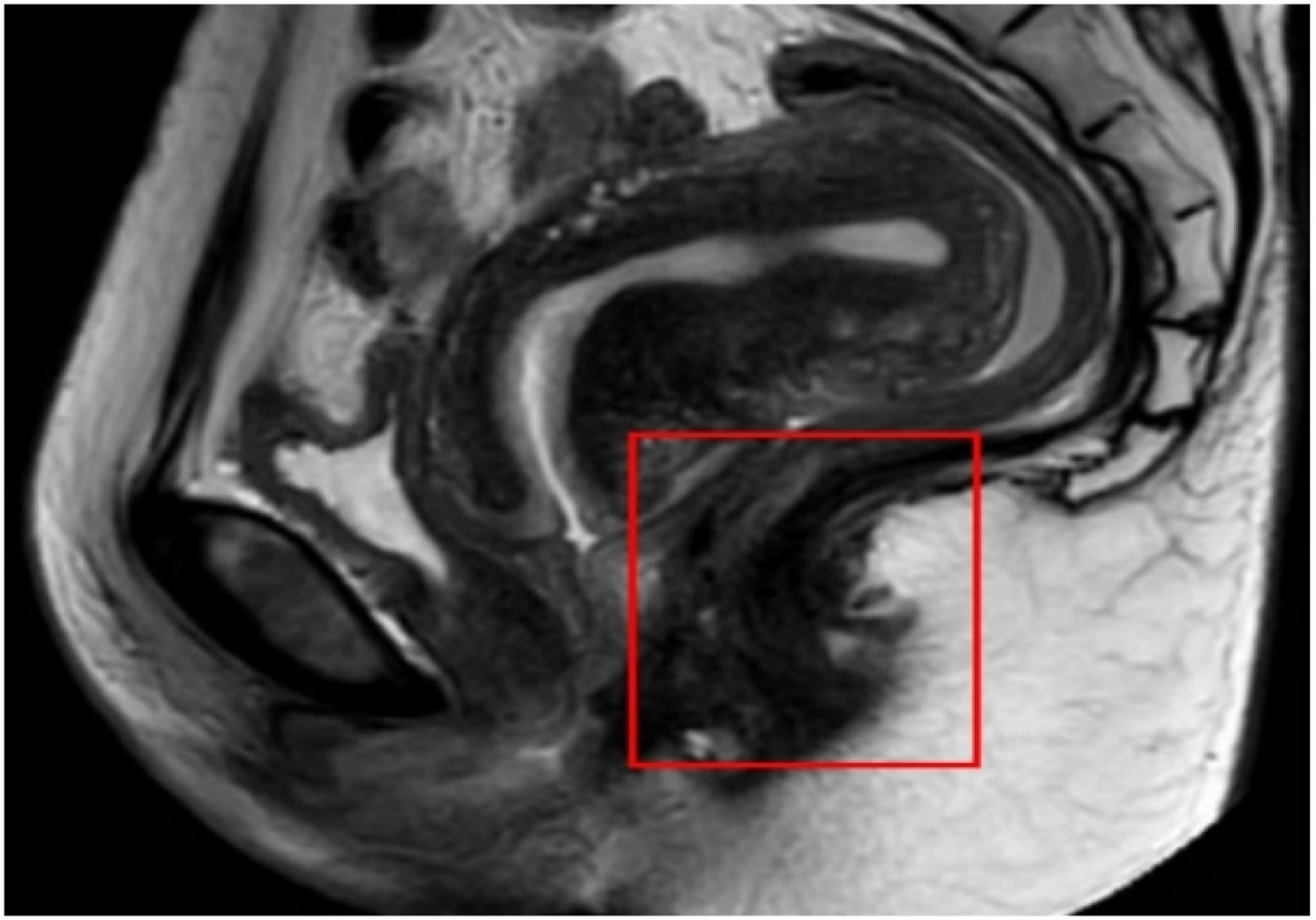
Pelvic MR images at 6 months after surgery (sagittal plane), the infection has resolved compared to previous imaging, with formation of a chronic fibrotic scar.

**Figure 6:**
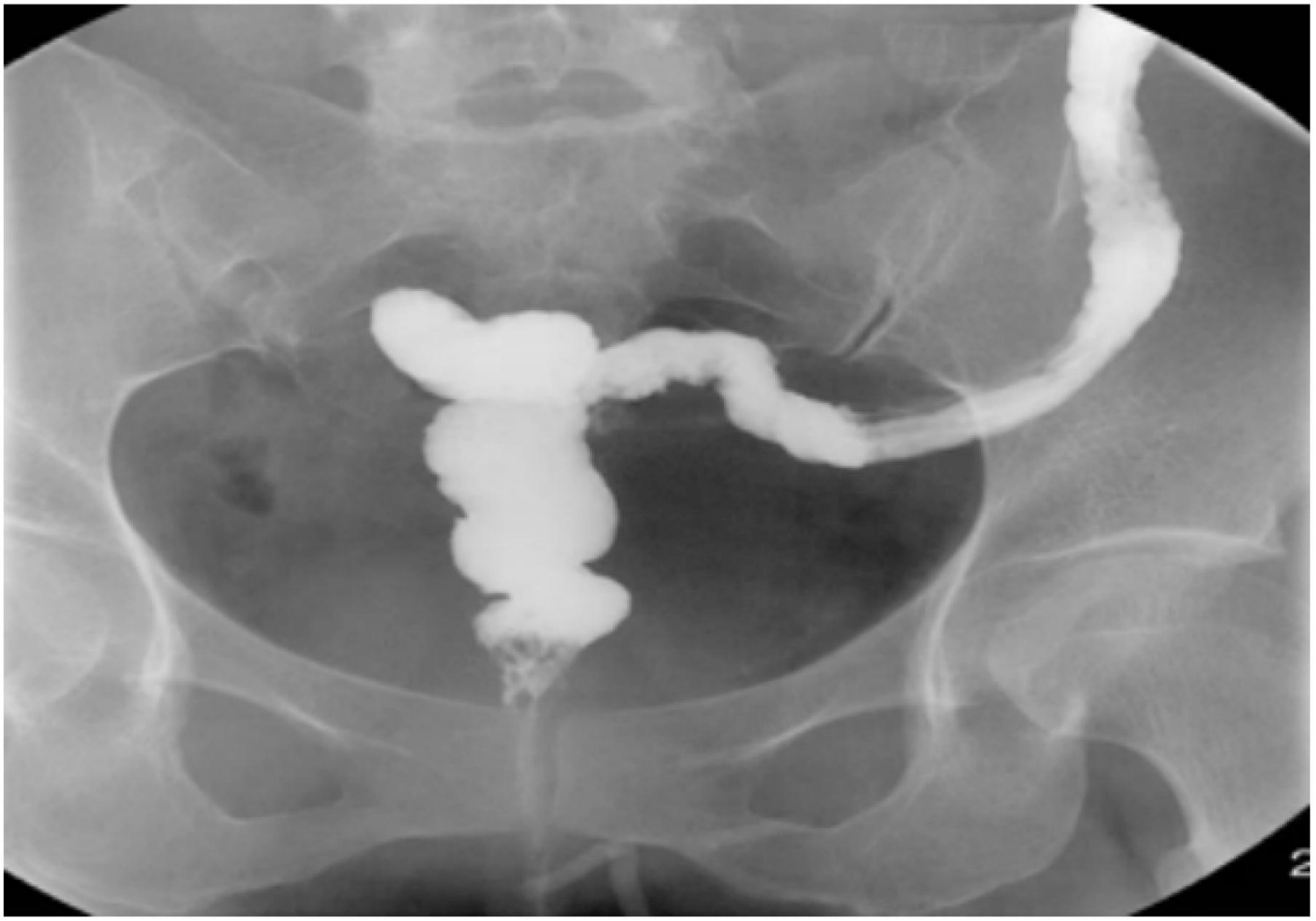
Defecography at 6 months, shows no contrast agent leakage, indicating complete healing of the fistula tract.

**Figure 7:**
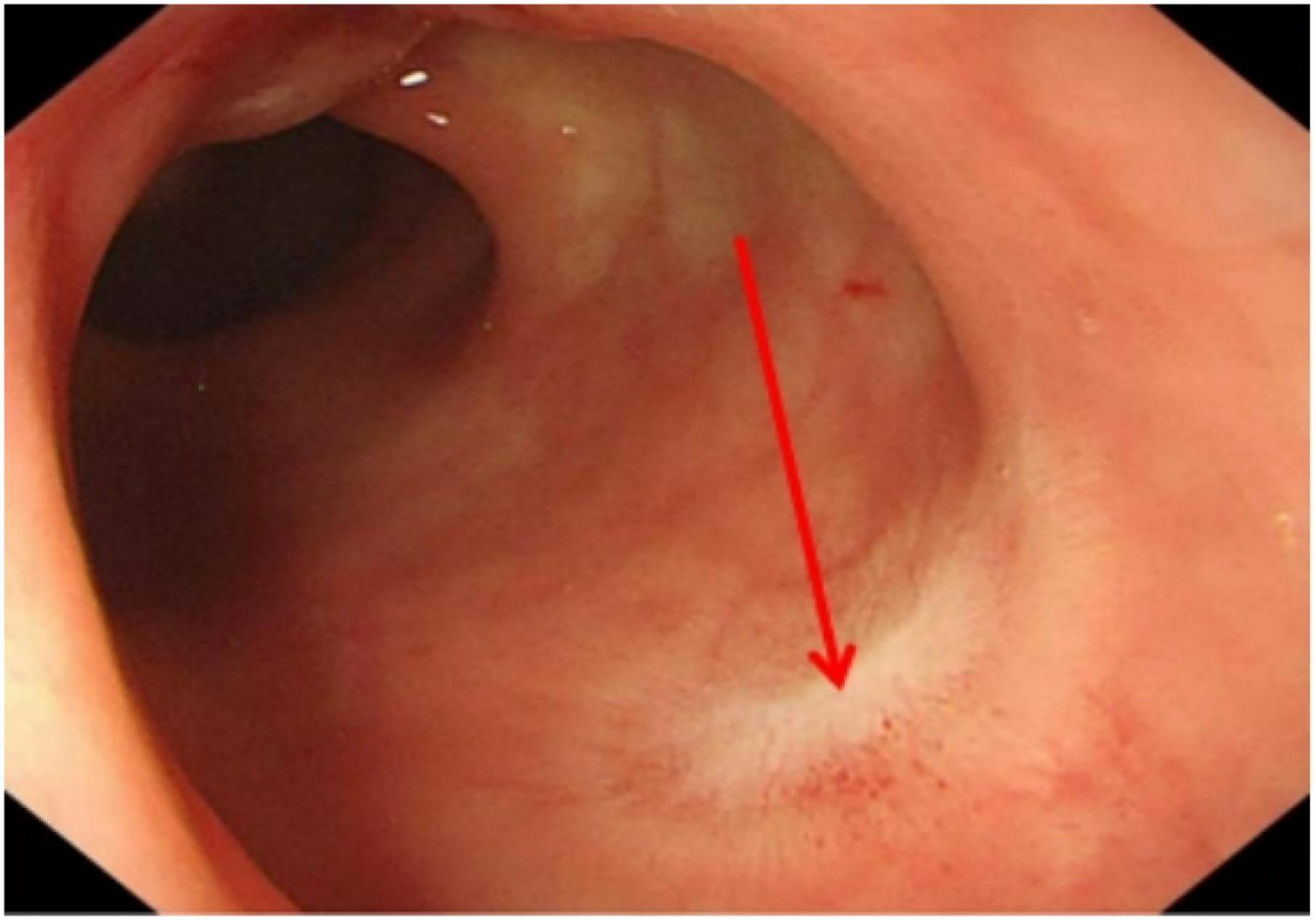
Colonscopy images at 6 months, shows no fistula opening; the arrow points to the scar formed after healing of the fistula.

## 3 Discussion

### 3.1 Strengths and limitations of the approach

The patient underwent laparoscopic double-barrel transverse colostomy to achieve fecal diversion, thereby preventing progressive infection caused by bacterial translocation. Although some reports have described primary repair or resection with anastomosis as treatment options for rectal perforation (7). Nevertheless, a phased surgical approach was eventually used because of the patient's extended illness, significant rectal wall edema, and concurrent presacral infection. Given the low location of the perforation, double-barrel transverse colostomy was selected. In order to achieve total fecal diversion, create a reasonably clean environment for rectal healing and infection control, and lay the foundation for later colostomy reversal, this stoma was placed distant from the infectious focus. The infected lesion interacted with the anal canal, creating a native drainage tract, and the fistulous orifice was situated 4 cm above the anal margin. Purulent fluid could leak into the anal canal when the patient was in the semi-recumbent position. In order to maintain unobstructed drainage at this point, the surgical team used transanal irrigation and intraoperative moderate dilatation of the rectal fistula. This prevented the need for additional drainage incisions in the sacrococcygeal region and decreased the risk of neurovascular injury and secondary infection (8). The colostomy maintained a feces-free environment in the distal bowel segment, allowing repeated evacuation of exudate and necrotic debris from the infected cavity, maintaining local cleanliness to facilitate tissue repair. The team planned transanal scar resection or rectal anastomosis since the scar hyperplasia at the mucosal defect in the latter stage may result in the linear stenosis of the rectal stenosis (9). The postoperative recovery of the patient was the better than anticipated, and no lower rectal stenosis occurred.

Nevertheless, there are inherent drawbacks to this surgical approach. Incomplete drainage from isolated transanal irrigation can result in recurring or persistent infections. Secondary reconstructive surgery will be required for fistula repair if the fistula does not heal after a long time. If the fistula fails to heal over an extended period, secondary reconstructive surgery will be necessary for fistula repair.

### 3.2 Discussion of relevant medical literature

RBL is a procedure that uses a rubber band to elastically ligate internal hemorrhoids, cutting off blood supply and causing tissue ischemia, necrosis, and sloughing. It is frequently used to treat internal hemorrhoids in stages I–III as well as grade IV situations in which patients have surgical contraindications or are unwilling to have surgery. Bleeding, pain, and dysuria are common complications, while severe complications such as refractory urinary retention, postoperative sepsis and rectal perforation are rarely documented in the literature (10). Sclerotherapy is generally performed within the submucosal layer (11). It induces vascular occlusion and tissue fibrosis to accomplish hemorrhoidal tissue shrinkage and hemostasis, and can be used in patients with both internal and mixed hemorrhoids. There have been reported cases of rectal perforation (12) and necrotizing fasciitis (13) following injection therapy.

In this case, the primary cause of the perforation was sclerotherapy, with hemorrhoid ligation serving as a contributing factor. In terms of the automatic hemorrhoid ligator's specifications, clinically common devices include a negative pressure system, suction component, and ligation unit. The diameter of the mucosal bulb created following ligation is typically 0.8–1.0 cm, and the negative pressure is regularly maintained at −0.08 to −0.09 kPa (14). Clinically, ligation is usually done 1–2 cm above the dentate line. Based on the patient's imaging results obtained after admission, which show the presence of a rectosacral fistula, it is possible that the ligation site may extend beyond the safe area above the dentate line and be located on the posterior wall of the rectum. At this location, the intestinal wall is weaker and lacks tissue protection, rendering it particularly vulnerable to full-thickness injury and consequences, including presacral infection. Rubber bands usually come off five to ten days later. Over this interval, the rectal wall is subjected to a prolonged state of ischemia. At this stage, if combined with sclerotherapy, the injection is not strictly restricted to the submucosa, and the agent may mistakenly penetrate the muscle layer. The sclerosant caused ischemia and necrosis in the already frail intestinal wall, which led to subsequent perforation and infection. If the ligation site is located on the anterior wall of the rectum, it may result in a rectovaginal fistula.

Both hemorrhoid ligation and sclerotherapy carry risks of perforation. However, cases of perforation induced by ligation alone are relatively rare. Based on clinical experience and literature review, sclerotherapy is considered the primary factor for perforation in this case, while ligation serves as a contributing factor. The ligation was placed too high and too deep, involving the rectal wall and resulting in ischemia and edema of local tissues. When combined with sclerotherapy, there was a risk that the needle would be inserted too deeply. The sclerosant would immediately cause ischemia and necrosis of the rectal wall tissue once it had reached the muscle layer. On the basis of ligation, sclerotherapy accelerated and aggravated the process of tissue necrosis, ultimately leading to perforation. Anal stenosis is one of the serious complications of hemorrhoid surgery (15). Postoperative edema, scar contracture from extensive mucosal resection, and sphincter spasm due to postoperative wound discomfort are all common reasons (16). In this case, the patient had circumferential “incarcerated hemorrhoids”. Extensive ligation caused substantial mucosal loss and elevated tension in the surrounding tissues. In the acute phase, inflammation generates edema in the local perianal tissues. Mild stenosis can be treated with dietary adjustments, stool softeners, or anal dilation. For patients in whom conservative treatment fails, scar release or flap plasty can be performed (9). Fecal diversion, primary perforation repair, presacral drainage, parenteral antibiotic treatment, and distal colonic lavage are the fundamental therapeutic principles for rectal perforation (17). The sigmoid and transverse colon are common sites of stoma, which can realize fecal diversion, prevent bacterial translocation and protect the terminal intestinal tube. Currently, no consensus has been achieved on the superior stoma position, which must be chosen by the operator based on his personal experience, condition, and postoperative difficulties (18). Because of the presacral region's distinct anatomical shape, indwelling drainage catheters are used to treat infection, prevent the formation of closed abscess chambers, and avoid secondary infection at the stoma (19). Drainage options include sacrococcygeal incision and tube placement (20) or image-guided puncture and tube placement by perineal (8) or transanal (12) route, with ongoing postoperative irrigation.

### 3.3 Take-away lessons

Rubber band ligation and sclerotherapyare invasive procedure. The standardized application of surgical techniques and careful preoperative patient selection are core prerequisites for reducing complications. It should be performed with the completely visualization of the operative filed,in strict accordance with standard operating protocols and surgical indications. Close attention should be paid to the patient's bowel preparation, and multiple ligations at a single site are not recommended. Mixed hemorrhoids are typically treated with external excision and internal ligation. External hemorrhoidectomy combined with internal hemorrhoid ligation and sclerotherapy is frequently adopted clinically to reduce intraoperative bleeding and postoperative pain for the patients with severe mixed hemorrhoids (21). However, the patient showed acutely incarcerated hemorrhoids upon admission, characterized by edematous and ischemic with indistinct tissue planes. Sclerotherapy at this period poses a significantly increased risk. Following the resolution of edema, an elective evaluation of surgical indications should be performed. Reviewing the clinical course of this case, the guidelines for sclerotherapy should be closely followed, and surgical options should be chosen with prudence.

The ligation site should be 1–2 cm above the dentate line, ligation should not be performed too high up. The suction pressure and ligation size of the automatic ligator are also important considerations; once suction reaches the “full red sign”, the ligation ring is released (22, 23). Keep the ligator's negative pressure between −0.08 and −0.09 kPa. The diameter of the mucosal bulb formed per ligation should vary from 0.6 to 1.0 cm; overly large bulbs should be avoided. Sclerosing agent should be injected into the submucosal layer, and injection of agents into the rectal muscular layer or mucosal epithelium should be avoided. Adequate preoperative risk stratification of patients is mandatory. Circumferential incarcerated hemorrhoids, high-located hemorrhoids, prior anal surgery, and chronic constipation are the predisposing factors for rectal perforation. Closely monitor the patient's vital signs, such as heart rate and blood pressure after surgery. An exploratory procedure should be carried out right away to ensure early intervention and treatment, thereby preventing the development of serious complications like sepsis or hemorrhagic shock, if the patient experiences massive postoperative bleeding, progressively worsening anal pain that cannot be relieved by medication, or systemic fever.

## 4 Summary

The patient received external hemorrhoidectomy combined with rubber band ligation and sclerotherapy. We hypothesize that the primary factor for perforation in this case is sclerotherapy, while rubber band ligation serves as a contributing factor. Given the low location of the perforation, a spontaneous drainage tract developed, and intraperitoneal infection was avoided. We used a double-barrel transverse colostomy to maintain complete fecal diversion and halt inflammatory progression, alongside transanal lavage to sustain unobstructed drainage; this combined strategy yielded favorable clinical outcomes. For patients who appear with low rectal perforations communicating with a presacral infectious cavity, the dual method of fecal diversion with transanal irrigation described here may be a useful therapeutic reference.

## Data Availability

The original contributions presented in the study are included in the article/Supplementary Material, further inquiries can be directed to the corresponding author.
